# Mapping the relationship of reef structure and surfer spatial patterns at Cloudbreak, Fiji

**DOI:** 10.1038/s41598-025-30878-6

**Published:** 2025-12-04

**Authors:** Clifford A. Kapono, Kailey H. Pascoe, Haunani H. Kane, Manuela Cortes, Sofia B. Ferreira, Makoa Pascoe, Aralyn Hacker, Fiona Ryan, Brianna K. Ninomoto, Kainalu K. Steward, Maluhia Stark-Kinimaka, Joseph W.P. Nakoa III, John H.R. Burns

**Affiliations:** 1 Center for Global Discovery and Conservation Science, School of Life Sciences, Arizona State University, Hilo, HI 96720 USA; 2 Marine Science, Data Science, and Tropical Conservation Biology and Environmental Science, College of Natural and Health Sciences, University of Hawai’i at Hilo, Hilo, HI 96720 USA; 3 Department of Earth Sciences, School of Ocean & Earth Science & Technology, University of Hawai’i, 1680 East West Rd, Honolulu, HI 96822 USA; 4 MEGA Lab, Hilo, HI 96720 USA

**Keywords:** Ecology, Environmental social sciences, Ocean sciences

## Abstract

This study examines how three-dimensional reef structure relates to surfer activity at KuruKuru Mailani, Fiji (Cloudbreak). We combined GPS surfer track data with structure-from-motion (SfM) photogrammetry to map surfer behavior on high resolution, orthorectified reef models. Surfer track density and cumulative track length differed significantly among three zones, with the highest activity in the Middle zone. Live coral cover did not differ among zones and showed no association with surfing activity. In contrast, three-dimensional structure varied by zone: VRM was higher in the Left than the Right, slope was smaller in the Left than in the Middle and Right, and surface complexity was higher in the Middle than the Left. A multivariate matching approach identified a combination of surface ruggedness, curvature, planform curvature, and surface complexity that best aligned with surfer activity. A PCA of these variables explained 39.8% and 24.7% of variance on the first two axes and showed that the Middle zone spanned the widest range of 3D values and the highest amount of surfer activity. Boosted regression trees indicated that surfer activity increases most within specific ranges of surface complexity and slope, with additional contributions from curvature and ruggedness. The partial dependence plots showed threshold-like responses rather than simple linear trends. Collectively, these results indicate that specific aspects and ranges of reef structure are most closely associated with the spatial concentration of surfer activity at Cloudbreak. The workflow presented here provides a consistent approach that can be applied at other surf breaks, and the findings offer clear directions for future studies to link structural features with wave processes and to inform management of recreational reef environments.

**Supplementary Information:**

The online version contains supplementary material available at 10.1038/s41598-025-30878-6.

## Introduction

Surfing ecosystems represent a unique socio-environmental system^1–3^. With approximately 50 million users globally making contributions over $60 billion USD to the economy each year^4,5^, surfing ecosystems serve as a valuable model for studying ocean recreation and tourism communities^6–8^. Surf breaks provide opportunities for leisure, artistic inspiration, cultural enrichment, and engagement with the environment^9^. Reef-building corals construct these reefs over thousands of years, creating surf breaks that sustain diverse ecosystems^10^. Coral reefs provice habitat to a significant proportion of marine species while also protecting coastal communities from erosion and storms^11–13^.

Among the services provided by coral reef ecosystems are surf breaks, which form where reef structures shape ocean wave energy into favorable surfing conditions; however, surf breaks also occur in non-tropical regions where rocky reefs, sandbars, or headlands create similar wave-shaping effects. The future of many reef-based surf breaks is increasingly threatened by the degradation of the coral reefs that form them. Climate change, coastal development, and reef loss can alter reef morphology and bathymetry. Such physical transformations not only disrupt ecological processes but can also diminish the quality and persistence of iconic surf breaks. Maintaining or restoring fine-scale topographic complexity is therefore critical to sustaining both the ecological integrity of reef systems and the cultural, recreational, and economic benefits associated with surfing ecosystems^2,9,14,15^.

Despite the ecological and social value of reef breaks used for surfing, there remains a notable gap in research focused on human interactions with these coastal features^3,10,16^. Since the pioneering studies on surf breaks in the early 1970 s, surf science has progressed, especially with advancements in coastal management, artificial reefs, and wave pool technologies^9,10,14,16^. Previous research has primarily focused on understanding the physics, engineering, and bathymetry of surf break waves. However, no studies to date have performed a detailed analysis of the three-dimensional (3D) reef structure and coral composition at surf breaks to determine whether surfing activity is localized around specific reef features, or whether surfing activity itself has any adverse effects on live coral cover. Further investigation is needed to examine how the 3D habitat structures of coral reefs may influence wave quality and how they relate to the spatial distribution of surfer activity.

Our study addresses these knowledge gaps by using high-resolution methods to investigate how 3D reef structures and coral cover relate to patterns of surfing activity across a reef break. Specifically, we examine whether surfing activity is localized around particular physical features of the reef and assess whether areas of high use correspond to differences in live coral cover. Although live coral supports reef biodiversity and resilience, its relationship to surfer use is unclear. Here we test whether variation in coral cover aligns with spatial patterns of surfing across the reef, and we evaluate how fine-scale three-dimensional geomorphology relates to surfer use intensity, clarifying which physical features are most associated with areas of concentrated surfer use.

Emerging technologies, such as GPS tracking devices and Structure-from-Motion (SfM) photogrammetry, offer new approaches to characterize reef structure and visualize recreational surfer interactions on reefs^7,16,17^. SfM photogrammetry has become a valuable tool for creating detailed 3D maps of marine environments, particularly coral reefs^18–21^. In coral reef research, SfM has been widely applied to quantify benthic cover, assess habitat complexity, and monitor changes in reef condition over time, making it an important method for ecological studies as well as human–reef interaction research. Unlike traditional bathymetric assessments, SfM photogrammetry produces high-resolution spatial data at the centimeter to millimeter scale, allowing subtle variations in reef structure to be captured and providing a more precise analysis of how physical attributes may influence wave formation and surfer activity. In addition to generating 3D reconstructions, this technique produces digital elevation models (DEMs) and orthophotomosaics, providing geospatial data for analyzing various ecological and physical attributes of reef landscapes^18,22^. By leveraging standardized, high-resolution methodologies, this study helps establish an analytical framework for future research examining how reef structure influences wave dynamics, surfing activity, and broader ecological interactions at surf breaks.

**Fig. 1:**
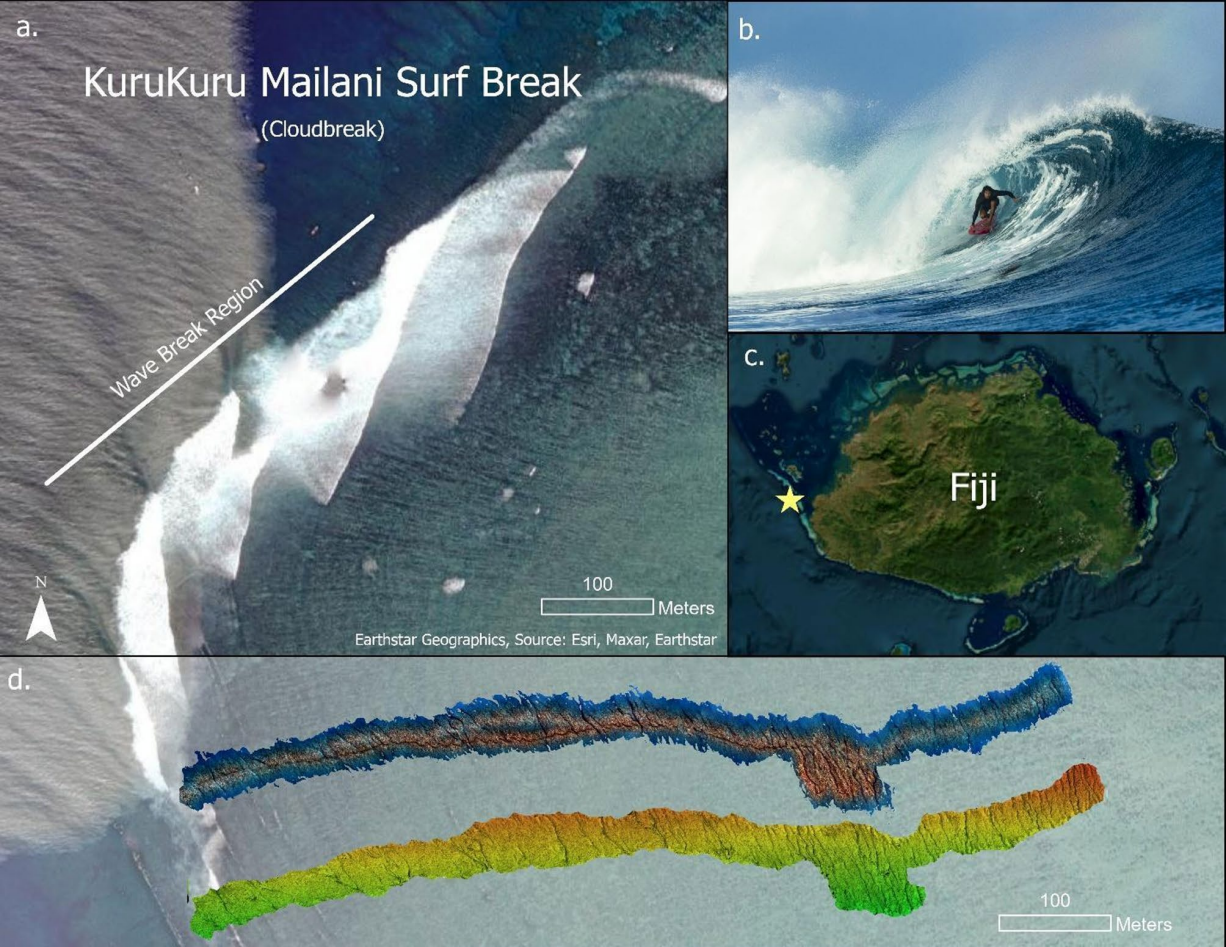
Overview of the KuruKuru Mailani Surf Break (Cloudbreak), Fiji. (**a**) Surf Break Region of KuruKuru Mailani, Fiji (also known as Cloudbreak). (**b**) Photo image of the world-class surf break at KuruKuru Mailani. (**c**) Map of Fiji, with a yellow star marking the location of the Cloudbreak surf break. (**d**) Orthophotomoasic (top) and Digital Elevation Model DEM (bottom) of the surf break region of KuruKuru Mailani (substrate depth ranged from approximately 1.5 to 4 m)

Our study site is KuruKuru Mailani, Fiji, known globally as “Cloudbreak” (Fig. 1). This surf spot is located approximately 5 km off the coast on the reef surrounding Tavarua Island, Fiji. Cloudbreak is recognized for its large, powerful waves that can reach heights of up to 10 m (about 30 ft) during major swell events, establishing its reputation as one of the world’s premier surf breaks^23^. Wave energy typically approaches the reef at an oblique angle from the south to southwest (210°–240°), with mean significant wave heights ranging from 2 to 4 m under typical conditions and exceeding 10 m during large winter swells^24,25^. These high-energy waves refract over a steep fore-reef and break along a shallow reef pass, producing long, barreling left-hand waves shaped by the underlying coral topography^23^. The reef structure directly influences wave formation, size, and quality, making Cloudbreak an ideal location to investigate the interactions between human activities, such as surfing, and the surrounding reef environment. Insights from this research have broader implications for coastal management, coral reef conservation, and the promotion of sustainable recreational practices.

## Materials and methods

### GPS tracking of surfer activity

To quantify spatial patterns of surfer activity, GPS data were obtained from Surfline, a surf forecasting and analytics company based in Huntington Beach, California. These data consisted of 9,704 anonymized surf session records collected in 2022 via smart watches worn by surfers, which captured GPS coordinates throughout individual wave rides; due to anonymization, unique surfers could not be distinguished. The spatial distribution of surfer tracks was analyzed using GPS-based surf tracking data to identify areas of concentrated activity, including take-off zones and ride trajectories. Mean track positions were calculated, along with the first and second standard deviations of their spatial distribution. We computed the mean start and end positions of rides and their first and second standard deviations. Using these statistics, we defined the primary analysis extent along the ride axis from one standard deviation before the mean start location to one standard deviation beyond the mean end location. We also calculated average ride length as the distance traversed on a wave between the recorded start and end points, so the analysis window corresponds to the core portion of distances typically covered during rides. This spatial extent was selected to capture the portion of the reef most frequently used during surf activity based on statistical thresholds rather than subjective placement. To further resolve the spatial structure of the break, we partitioned the reef into three zones arbitrarily named Left, Middle, and Right. The Left zone is nearest the predominant initiation of surfer tracks, the Right zone is nearest the predominant completion, and the Middle zone is the segment farthest from both initiation and completion.

This study did not involve human subjects, human tissue, or personally identifiable information. GPS tracking data used to quantify surfing activity were collected by Surfline, Inc., through voluntary use of GPS-enabled smart devices by surfers. All data were fully anonymized prior to access and analysis by the research team. The dataset included only geospatial coordinates corresponding to surfing activity (wave start and end points) and did not contain any demographic or health-related information. All methods were carried out in accordance with relevant guidelines and regulations pertaining to the use of anonymized spatial data.

### Three-dimensional photogrammetry survey

Structure-from-Motion (SfM) photogrammetry was used to generate high-resolution three-dimensional (3D) models of the Cloudbreak reef habitat within the area of concentrated surfing activity (located at −17.888116 S, 177.185108 E). GPS based surf track data were used to delineate the portion of the reef with the highest concentration of surfing activity. The zone definitions and spatial statistics described above were then used to set the spatial extent for the SfM mapping. To account for variability in surfer ride lengths, the mapped area extended further “up” the reef into the left zone, where several outliers indicated longer rides originating earlier along the reef, likely associated with larger swell events producing longer-breaking waves. In contrast, the end point of the right zone was more distinct, as waves typically terminate consistently in a deeper channel within the reef structure. This mapping approach ensured that the habitat surveys encompassed the full spatial range of active surfing areas, enabling analysis of how specific reef features influence patterns of surfer use. The swath width for SfM image collection was determined using the density of GPS surfer tracks, with survey boundaries adjusted to exclude outlier tracks observed during larger swell events and to avoid areas that were too deep or too shallow for reliable reconstruction. The SfM technique uses overlapping two-dimensional images to generate a 3D model of the coral reefs community. Images were collected by a diver in June 2023. Images were taken from a planar angle approximately 2 m above the substrate in a boustrophedonic (lawn mower) pattern with 70–80% overlap between images using a Sony a7rIII camera with a 14 mm rectilinear lens. A total of 16,361 images were collected by the diver during the survey effort. Scale bars with coded targets were placed throughout the survey area to enable orthorectification of the resulting 3D reconstructions. 3D reconstructions were rendered using Agisoft Photoscan/Metashape Professional software (Agisoft LLC., St. Petersburg. Russia) following the methods described in Burns et al. 2015^18^. The software used the coded targets on the scale bars to assign reference points to orthorectify the models to a known local coordinate system. To minimize warping and distortion, self-calibrating bundle adjustments were completed using the known reference points to refine both camera positions and lens parameters. This optimization step corrected for geometric errors caused by underwater light refraction and camera lens distortion, ensuring that scale and geometry were preserved throughout the model. After optimization, a dense point cloud and solid triangulated mesh were rendered, from which an orthophotomosaic and a Digital Elevation Model (DEM) were exported to facilitate geospatial analysis and quantification of live coral cover.

### Assessment of reef structural Complexity, coral Composition, and surfer track patterns

**Fig. 2:**
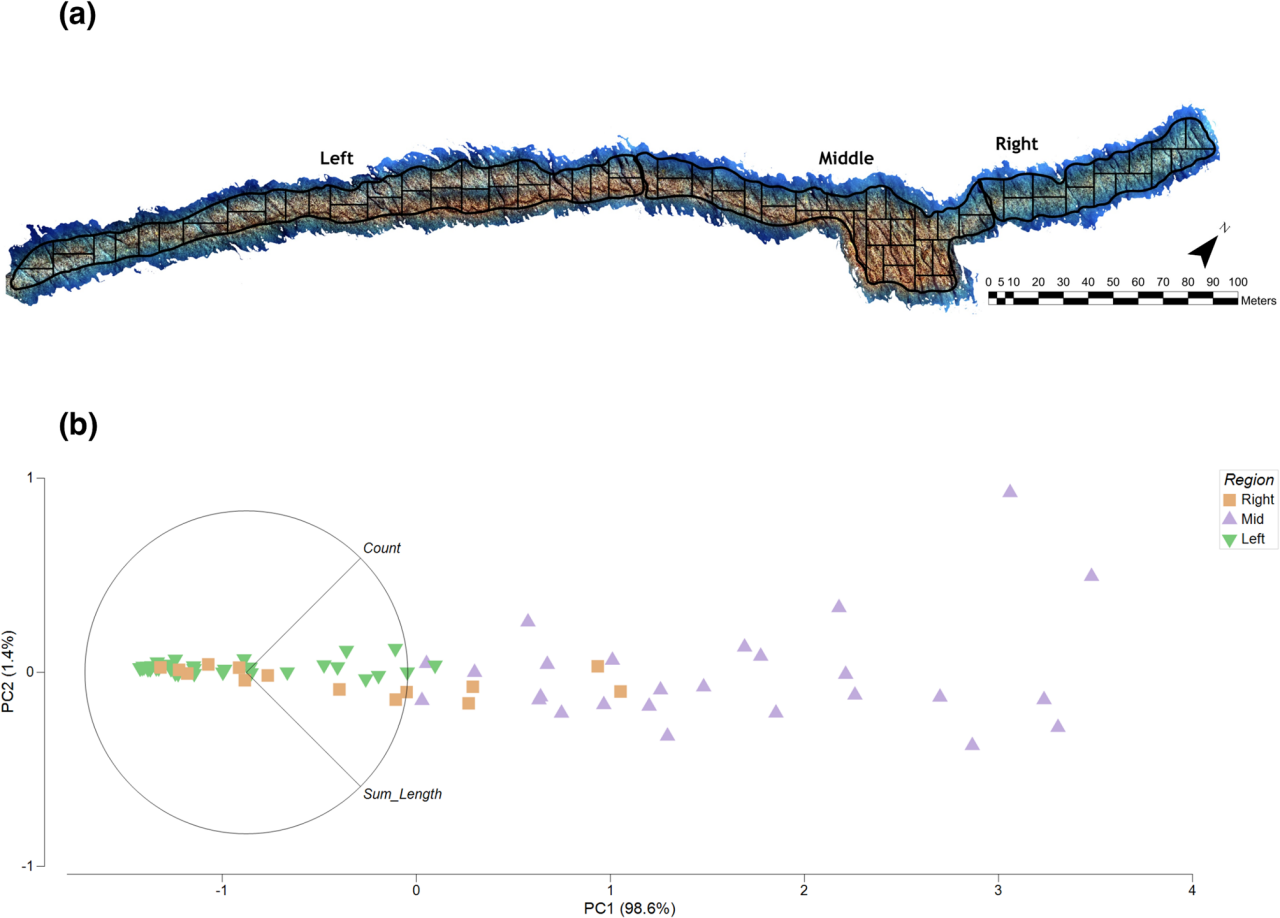
(**a**) Three-dimensional Model of KuruKuru Mailani, Fiji. The Left, Middle and Right labels depicted the different sections of the wave. Each reef zone was subdivided into 100m^2^ grids, resulting in 15 grids for the Right zone, 27 for the Middle, and 34 for the Left. (**b**) Principal Component Analysis (PCA) biplot of normalized surfer activity metrics (track count and cumulative track length) for each 100 m² grid, colored by reef zone. PC1 and PC2 explain 98.6% and 1.4% of the variance in the dataset, respectively. PC1 strongly reflects cumulative track length and serves as a one-dimensional summary of spatial variation in surfing activity across the reef

The DEM was exported at a 1 cm raster cell resolution, which is an established scale for quantifying fine-scale reef structure^18,22^ and was well within the range of model accuracy (i.e. millimeter-level accuracy) as reported in the results by the ground sampling distance and Root Mean Squared Error (RMSE) values obtained from ground control points (i.e. objects/features of known distances/dimensions) in the models. The 1 cm resolution DEM was subdivided into 100 m^2^ grids using the “Divide a Polygon by a Value” tool in GISPro software, resulting in 14 grids for the Right zone, 26 for the Middle, and 37 for the Left (Fig. 2). 3D metrics of structural complexity were calculated for each 100 m² grid across the three reef zones (Left, Middle, and Right; Fig. 2). Specifically, vector ruggedness measure (VRM), slope, curvature, planform curvature, profile curvature and surface complexity were computed in R Software using the raster^26^, sf^27^and rgeos^28^ packages, following the procedures outlined by Fukunaga et al. (2019)^21^. Metrics were derived using a moving window analysis with 3 × 3 cell neighborhoods across each 100 m² grid, generating a mean value and associated variance for each structural complexity metric. These metrics quantify different aspects of reef structure: VRM captures the degree of surface ruggedness, slope measures the steepness of the terrain, and profile curvature assesses the concavity or convexity along the direction of maximum slope, which can influence hydrodynamic conditions and habitat heterogeneity.

Within each reef zone (Left, Middle, and Right; Fig. 2), digital 4 × 4 m quadrats were randomly placed to subsample the reef habitat to quantify benthic composition. A total of 84 quadrats were positioned on the orthophotomosaic and exported for analysis using the CoralNet platform (coralnet.ucsd.edu). The quadrats sampled about 17% of the substrate in each zone, for a total of approximately 51% of the total mapped reef area. For each quadrat, 400 random annotation points were generated and classified to quantify the percent cover of live coral, algae, and abiotic features. Annotation points falling on transect tapes, scale bars, or mobile fauna were excluded from the analysis. All annotations were performed by trained individuals and independently checked for quality by additional team members. The high resolution orthomosaic imagery enabled clear delineation of live coral, ensuring identification accuracy.

We assessed associations between surfing activity and live coral cover by using GISPro to quantify the number and cumulative length of surf tracks intersecting each 4 × 4 m quadrat across all 84 quadrats. Associations between surfing activity and 3D reef complexity were examined by using GISPro to quantify the number and cumulative length of surf tracks within each 100 m² grid cell. Surf tracks were defined as the paths of surfers passing through each grid cell; however, not all tracks fully traversed a plot. To account for partial crossings, the sum of both counts and distance were calculated for each individual 100 m^2^ grid (Fig. 2, Fig. S1).

### Statistical analysis

We tested differences in mean live coral cover among zones using a one-way ANOVA on quadrat-level percent cover in R (v4.3.0; R Core Team). We also performed nonparametric bootstrapping with 1,000 resamples within each zone to generate confidence intervals and evaluate whether zone means differed. We compared reef zones using nonparametric Kruskal–Wallis tests with Dunn-type pairwise comparisons for both surfer activity metrics and 3D habitat metrics in R (v4.3.0; R Core Team). Multivariate structure in surfer activity was summarized with a PCA in PRIMER-e on normalized surfer variables. The first principal component (PC1) was retained for subsequent analyses^29^. To relate 3D metrics of reef structure to surfer activity, we applied the BEST (BIO-ENV) approach in PRIMER-e using normalized 3D metrics (mean slope, mean VRM, mean curvature, mean profile curvature, mean planform curvature, surface complexity). BEST evaluates, for all subsets of environmental variables, a Euclidean distance matrix and its rank correlation with the resemblance matrix of the normalized surfer data, identifying the subset that best correlates with the surfer activity data^30^. After identifying this subset, we ran a PCA in PRIMER e on the selected variables to visualize multivariate structure and interpret how their joint variation relates to surfer activity. Lastly, we modeled the association between 3D metrics of reef structure and surfer activity with a boosted regression tree approach in R, using the six 3D metrics as predictors and surfer PC1 as the response (Gaussian distribution, tree complexity = 2, learning rate = 0.001, bag fraction = 0.75, seven-fold cross-validation). Following Elith, Leathwick & Hastie (2008)^31^; we simplified the model, refit after dropping mean curvature, and interpreted relative influence and partial dependence from the fitted model.

## Results

### 3D reconstruction of reef habitat

The Structure-from-Motion (SfM) photogrammetry model of the Cloudbreak surf break exhibited a spatial error of 0.00165 m and a ground resolution of 1.09 mm per pixel, indicating high geometric precision. Model alignment produced a reprojection error of 1.46 pixels, consistent with established standards for high-resolution reef modeling. The final reconstruction covered an area of 8,919 m^2^, capturing a substantial portion of the surf break’s reef habitat.

### Comparison of surfer Activity, coral Cover, and reef structure

**Fig. 3:**
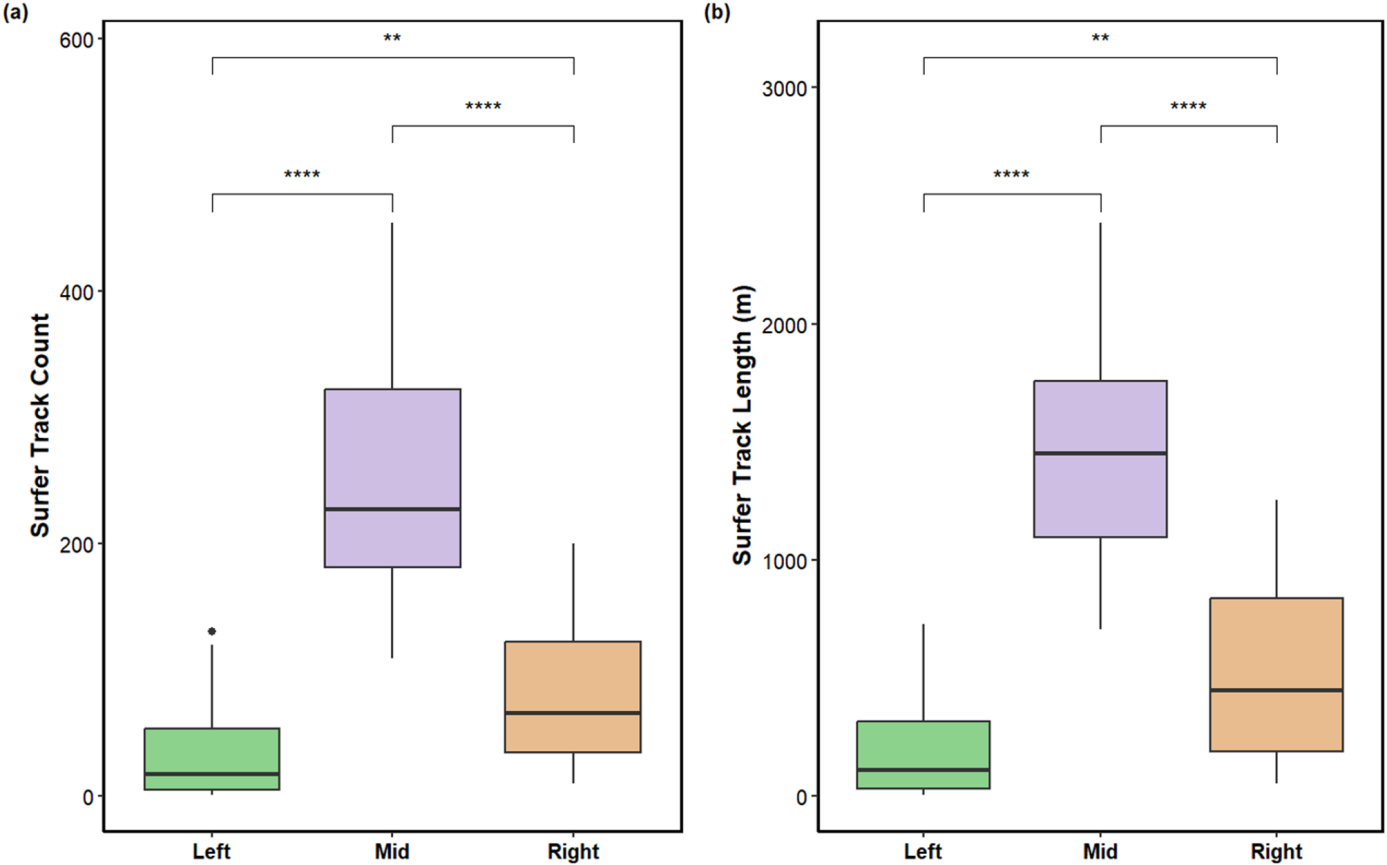
Surfer activity by reef zone. (**a**) Track count per 100 m² tile and (**b**) cumulative track length (m) per 100 m² grid for the Left, Mid, and Right zones. Brackets indicate Kruskal–Wallis pairwise comparisons (** *p* < 0.01; **** *p* < 0.0001)

Surfer activity, measured by track count and cumulative track length, was highest in the Middle zone and significantly greater than in both the Left and Right zones (Kruskal–Wallis, *p* < 0.001; Fig. 3). Pairwise comparisons also showed greater surfer activity in the Right zone than in the Left zone (*p* < 0.05). Tracks were most concentrated in the Middle zone, corresponding to the central section of the mapped surf break. Principal component analysis (PCA) of the normalized surfer activity identified two axes explaining 98.6% (PC1) and 1.4% (PC2) of variance, respectively (Fig. 2). Given that PC1 captures nearly all variation, it serves as a concise univariate index of surfer activity and is used in subsequent analyses.

**Fig. 4:**
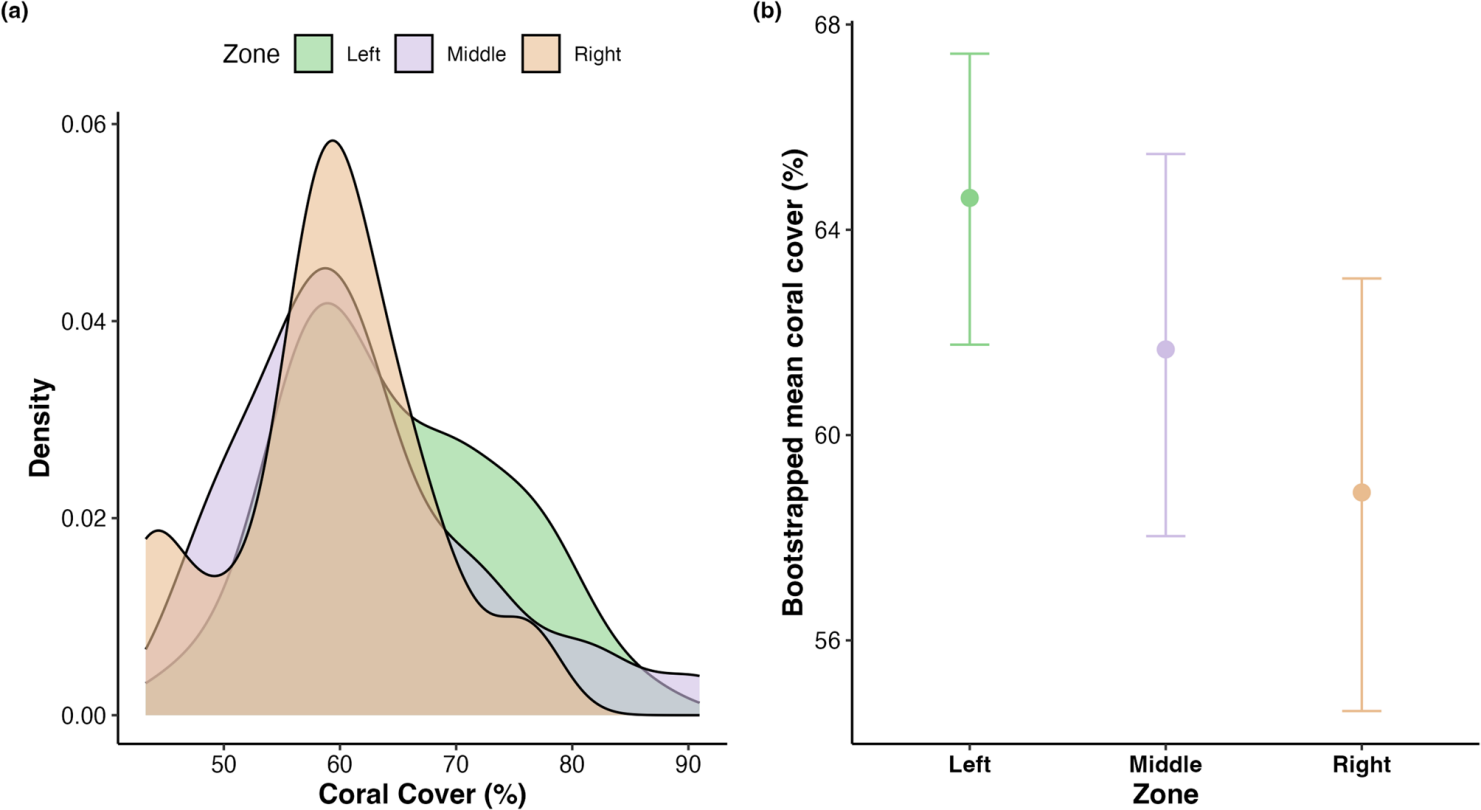
Comparison of live coral cover among reef zones at Cloudbreak, Fiji. (**a**) Density plots showing the distribution of percent live coral cover across the Left (green), Middle (purple), and Right (orange) reef zones. (**b**) Bootstrapped mean coral cover (± 95% confidence intervals) for each reef zone based on 1,000 resamples per zone. Mean coral cover was highest in the Left zone (64.6%), followed by the Middle (61.7%) and Right (58.9%) zones, with overlapping confidence intervals indicating no significant differences among zones (ANOVA, *p* > 0.05)

Mean live coral cover was highest in the Left zone (64.6% ± 9.32), with similar values in the Middle (61.7% ± 10.6) and Right (58.9% ± 8.71). A one-way ANOVA detected no significant differences among zones (*p* > 0.05; Fig. 4). Nonparametric bootstrapping (1,000 resamples per zone) yielded 95% confidence intervals of 61.8–67.4% for the Left, 58.0–65.5% for the Middle, and 54.6–63.1% for the Right, and the broad overlap among intervals corroborates the lack of a statistically significant difference. Overall, coral cover was relatively consistent across zones, and all zones supported moderately high live coral. No significant correlations were detected between coral cover and either track count or ride length, overall or within individual zones (Pearson’s r, *p* > 0.05).

**Fig. 5:**
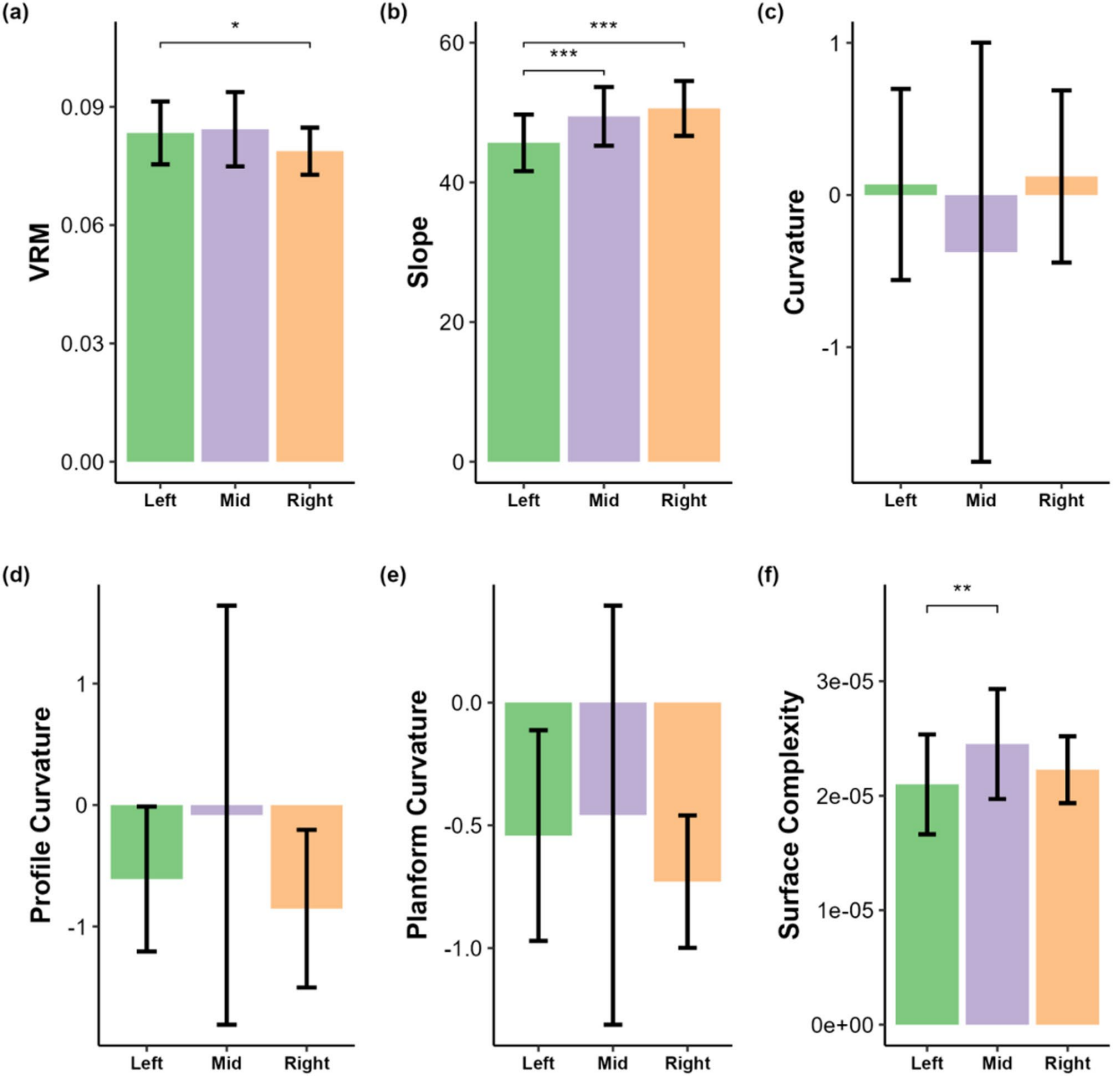
3D habitat metrics by reef zone. Bars represent the central tendency with error bars. Brackets indicate Kruskal–Wallis with Dunn pairwise results (* *p* < 0.05, ** *p* < 0.01, *** *p* < 0.001). (a) VRM was higher in the Left than the Right zone. (b) Slope was lower in the Left than in both the Middle and Right zones. (f) Surface complexity was higher in the Middle than the Left zone. Curvature metrics (c overall, d profile, e planform) did not differ significantly among zones, although the Middle zone exhibited a wider range of curvature values consistent with more alternating concave and convex features

3D habitat metrics differed among zones (Kruskal–Wallis with Dunn pairwise). VRM was higher in the Left than the Right zone (Fig. 5a; *p* < 0.05). Slope was lower in the Left than in both the Middle and Right zones (Fig. 5b; *p* < 0.001). Surface complexity was higher in the Middle than the Left zone (Fig. 5f; *p* < 0.01). Mean, profile, and planform curvature did not differ significantly among zones (Fig. 5c–e; *p* > 0.05), although the Middle zone exhibited a much wider range of curvature values, consistent with more alternating concave and convex features (e.g., spurs and grooves).

### BEST (BIO-ENV) correlation and PCA of surfer activity with 3D metrics of reef structure

**Fig. 6:**
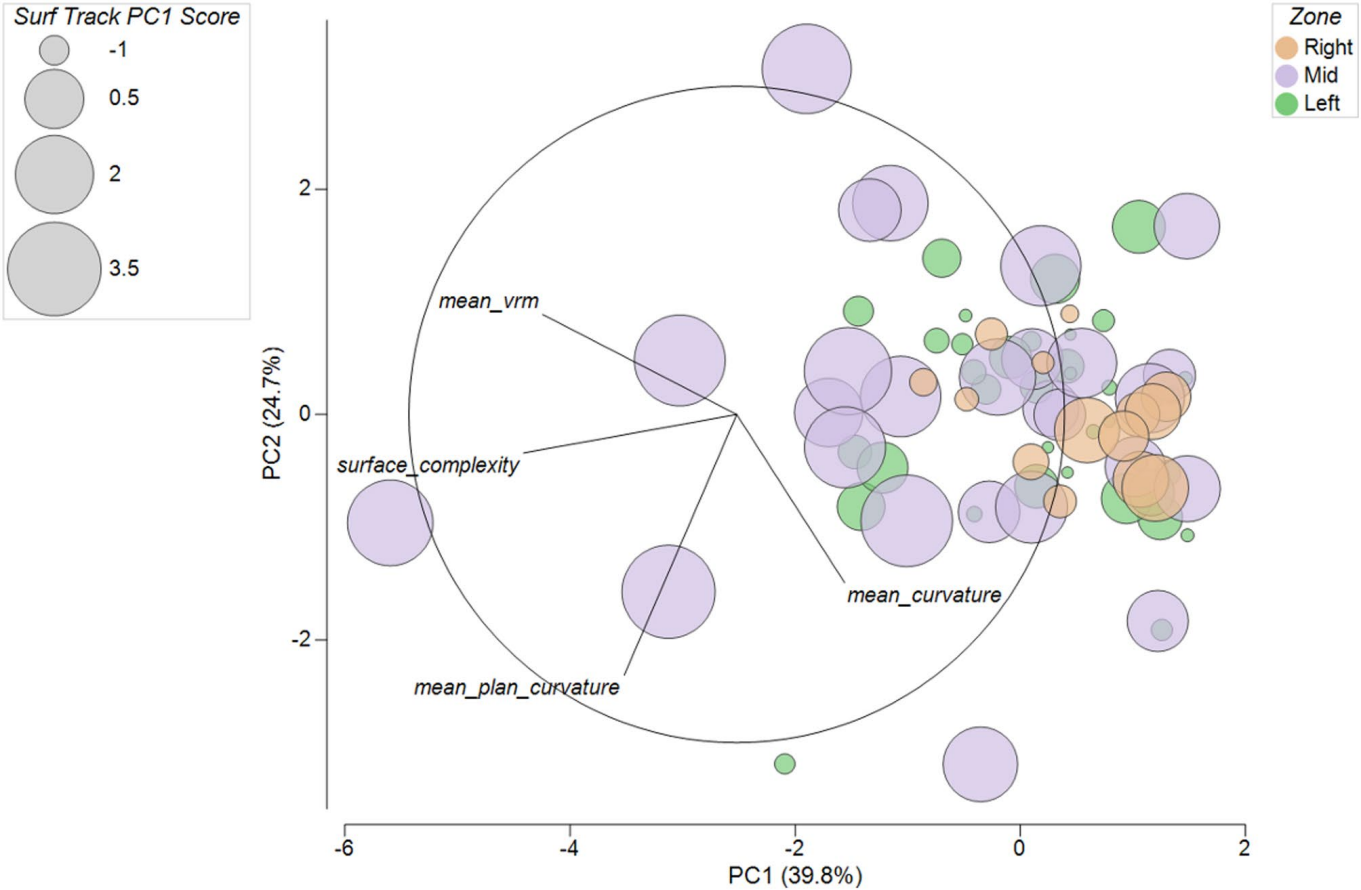
PCA biplot of selected 3D habitat metrics from the BIO-ENV/BEST subset (mean VRM, surface complexity, mean curvature, mean planform curvature). Points represent 100 m² grid cells colored by reef zone, bubble size indicates surfer activity PC1 score. Arrows show variable loadings. Axes display variance explained, PC1 = 39.8% and PC2 = 24.7%. Grid cells from the Middle zone span a wider range on both axes and often include larger bubbles, suggesting greater dispersion in 3D habitat structure and higher levels of surfer activity

The BEST (BIO-ENV) procedure identified mean VRM, mean curvature, mean planform curvature, and surface complexity as the subset of 3D metrics that best aligned with the surfer resemblance matrix data (PC1 of surfer activity), with ρ = 0.297 (*p* = 0.01 based on 99 permutations, and all randomized correlations lower than 0.297, Fig. S2). A PCA of this subset explained 39.8% (PC1) and 24.7% (PC2) of the variance (Fig. 6). VRM and surface complexity loaded most strongly on PC1, while planform curvature and overall curvature loaded most on PC2. Grids in the Middle region span the widest range along PC2 and show the largest bubbles, indicating higher surfer PC1 scores. This pattern suggests that areas with more extreme curvature variability, reflecting dynamically concave and convex structure, coincide with greater surfer activity (Fig. 6).

### Boosted regression tree (BRT)

**Fig. 7:**
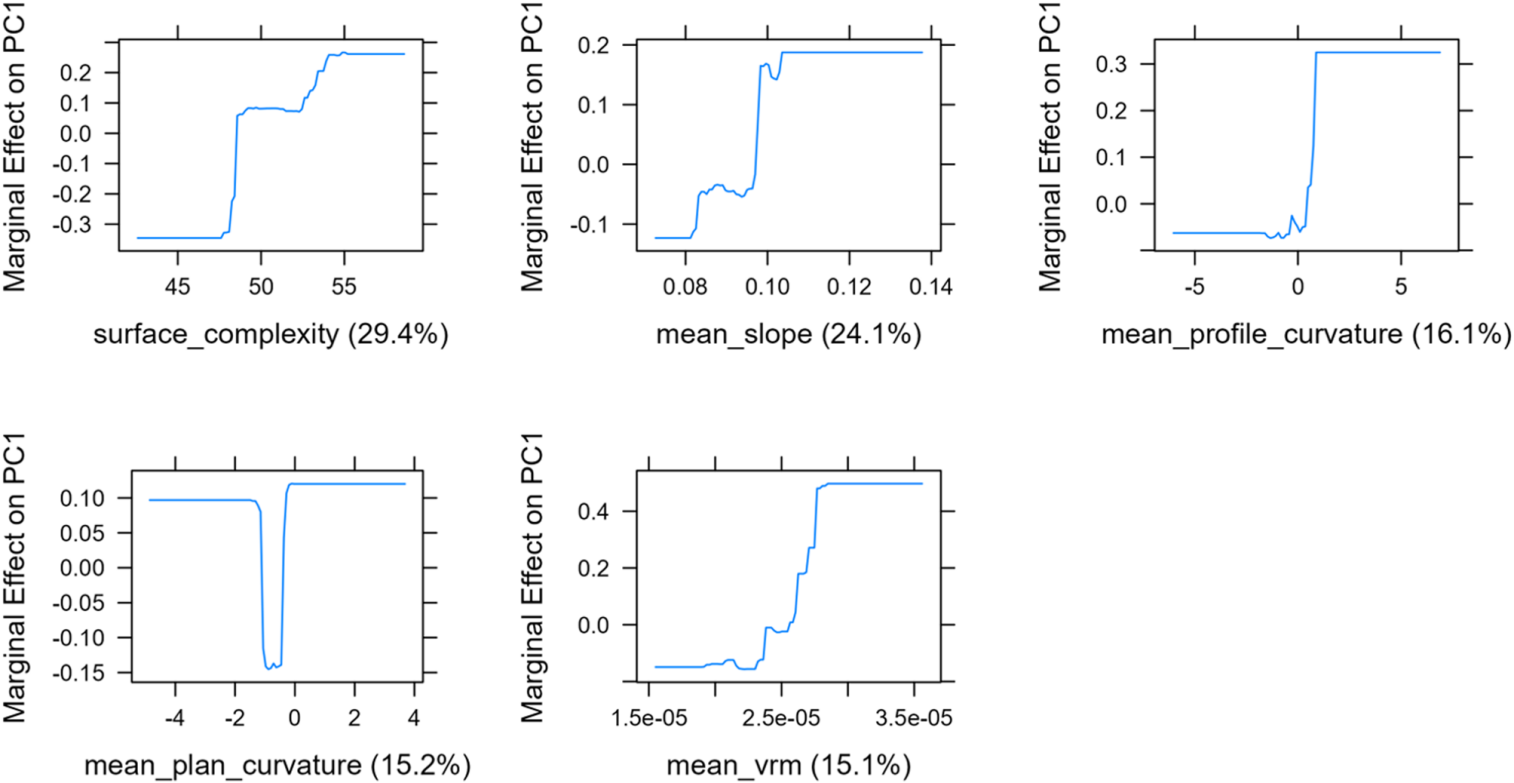
Partial dependence plots from the cross-validated boosted regression tree relating 3D habitat metrics to surfer activity as represented by PC1. Each panel shows the marginal effect of a predictor on PC1; percentages in parentheses are relative influence. Surface complexity, mean slope, and mean VRM show threshold-like positive responses. Mean profile curvature increases PC1 at more positive values. Mean planform curvature shows a shallow nonlinear trough near zero. Axes are on the original metric scales and effects are centered for comparison

The cross-validated BRT relating 3D metrics to surfer PC1 identified five predictors after model simplification (mean curvature dropped). Relative influence was surface complexity 29.4%, mean slope 24.1%, mean profile curvature 16.1%, mean planform curvature 15.2%, and mean VRM 15.1%. Partial-dependence plots (Fig. 7) show positive, threshold-like increases in PC1 with greater surface complexity, slope, and VRM; profile curvature is associated with higher PC1 at more positive values; planform curvature exhibits a weak, nonlinear effect with a shallow trough near neutral values. Together, these results indicate that grids with rougher, steeper, and more variably curved seafloor tend to align with higher surfer activity, while emphasizing association rather than causation.

## Discussion

To our knowledge, this is the first high resolution spatial characterization of surfer use on a heavily visited coral reef, KuruKuru Mailani, Fiji, commonly known as Cloudbreak. By combining GPS tracking data with SfM photogrammetry, we demonstrate an effective approach for mapping both human activity and reef habitat structure across coral reef environments. We focused exclusively on Cloudbreak to examine associations between reef structure and surfer activity at a location renowned for its energetic wave environment. While no traditional control reef was included, this targeted approach allowed for a detailed characterization of the physical and biological features that define one of the most dynamic surf reefs in the Pacific. The results demonstrate the effective use of GPS tracking devices as a proxy for human presence across remote reef environments. Moreover, surfers offer a unique window into human–environment interactions through the lens of ocean recreation, providing an underutilized but valuable dataset for studying spatial use in coastal ecosystems.

A key outcome of this research is the validation of GPS-enabled surf tracking data as a critical tool for spatially accurate mapping of human activity on coral reefs. In the case of Cloudbreak, GPS data were essential for identifying the specific sections of the reef actively used by surfers, allowing targeted habitat mapping that would otherwise be infeasible. Direct mapping of the reef during periods of active wave conditions is not possible due to the hazards of rough water and the need for calm conditions during photogrammetric surveys. Thus, surfer-provided GPS data created an ideal framework for spatially linking reef habitat features to patterns of human use. Unlike other ocean recreation activities, such as SCUBA diving or snorkeling, where GPS data are typically unavailable due to underwater signal limitations, surfing presents a unique opportunity for passive data acquisition. Surfers frequently utilize GPS-enabled smart devices and voluntarily upload their data to platforms like Surfline, driven by an interest in performance analytics such as speed, maneuverability, and ride trajectory. This practice overcomes major logistical challenges common in marine recreation studies and enables the collection of high-quality, crowd-sourced spatial data. The willingness of surfers to share geospatial information highlights the potential for citizen science to guide detailed scientific investigations of human–environment interactions on dynamic reef ecosystems (Supplementary Fig. S2).

The high-resolution 3D photogrammetric model of Cloudbreak provided an accurate spatial framework for investigating associations between reef structure and human activity. By aligning the reef model with mapped surfing activity, this study offers insight into how underlying habitat features and quantified reef structure relate to patterns of surfer use. Globally, many surf breaks overlap with areas of significant biological diversity and ecological value, making such studies important for understanding the interplay between human recreation and marine ecosystems^9,14^. At Cloudbreak, surfer track density and ride length were significantly higher in the Middle zone compared to the Left and Right zones (Kruskal-Wallis, *p* < 0.001; Fig. 3), with track density measured as the number of tracks per unit area. This concentration of activity corresponds to the central section of the surf break, which was strategically targeted for mapping based on GPS tracking data from previous swell events (Fig. 1). Consistent with this pattern, the PCA of surfer activity showed the Middle zone exhibits the highest PC1 scores, while the Left and Right zone cluster at lower values (Fig. 2). This justified focusing our analyses on how local structure varies across the zones of this surf break and whether specific structural conditions align with where surfers concentrate. While direct measurements of wave energy or swell characteristics were not collected, the observed spatial clustering of longer rides and greater track counts in the Middle zone supports the inference that this section of the reef provides more favorable and consistent surfing conditions. These spatial patterns highlight the potential for integrating 3D habitat mapping and recreational use data to better understand the environmental and geomorphological factors that contribute to wave quality at reef-based surf breaks.

Although the Left zone exhibited the highest mean live coral cover (64.6%), followed by the Middle (61.7%) and Right (58.9%) zones, a one-way ANOVA detected no significant differences among zones (*p* > 0.05; Fig. 4). Bootstrap 95% confidence intervals overlapped broadly (Fig. 4) and coral cover was not correlated with surfer activity. These results suggest that biological attributes such as coral abundance are not associated with the spatial distribution of surfer activity at Cloudbreak. These findings challenge assumptions that biological attributes, such as coral cover, are central to shaping recreational use of reef ecosystems^13,32^. Furthermore, the consistently high levels of live coral across all zones indicate that intensive surfing activity and high wave energy do not appear to have clear detrimental effects on live coral within the surveyed area. This finding highlights the potential resilience of live coral to withstand intense hydrodynamic disturbances and may offer useful insights into coral communities exposed to frequent high-energy wave conditions. Previous studies have shown that wave loading can shape coral community structure and favor growth forms adapted to withstand hydrodynamic forces, such as branching and massive morphologies^33^, suggesting that the coral assemblages at Cloudbreak may be naturally optimized for persistence under frequent wave-driven disturbance. Building on these findings, future research can leverage the spatial framework and ecological insights presented here to better investigate coral resilience and adaptation in surf-exposed reef environments.

The three-dimensionality of a reef’s shape strongly governs how waves shoal, refract, and break on reef platforms. Local slope influences shoaling rate, breaker type, and peel angle, with steeper faces promoting earlier and steeper breaking of waves that can organize takeoff zones and ride lines^34^. Surface roughness and texture, captured by VRM and surface complexity, modulate bottom friction and turbulence, which in turn affect energy dissipation and the shape of the wave face across the reef flat and fore reef^34^. Curvature terms describe how the surface bends and therefore how wave fronts are focused or dispersed. Profile curvature highlights channels and ridges that guide flow landward, while planform curvature captures side to side undulations characteristic of spur and groove fields that can steer refraction patterns along the crest and help set takeoff and trim zones^35^. Together these metrics quantify the geomorphic controls that are most often linked to wave formation and quality at surf breaks.

In contrast to coral cover, physical habitat metrics showed clear differences among zones. Mean VRM was greater in the Left than the Right zone (Kruskal–Wallis, *p* < 0.05; Fig. 5a). Mean slope was smaller in the Left than in both the Middle and Right zones (*p* < 0.001; Fig. 5b). Surface complexity was greater in the Middle than the Left zone (*p* < 0.01; Fig. 5f). Curvature means did not differ among zones, but the Middle exhibited a larger range of curvature values than the other zones (Fig. 5c–e). To relate reef structure to surfer activity, we first applied the BEST (BIO-ENV) procedure in PRIMER. This multivariate matching test identified a combination of mean VRM, mean curvature, mean planform curvature, and surface complexity that best aligned with surfer activity (rank correlation ρ = 0.297, *p* = 0.01 (99 permutations), Fig. S2). We then visualized these variables with a PCA. The first two components explained 39.8% and 24.7% of the variance in surfer activity, with VRM and surface complexity loading most on PC1 and planform curvature and overall curvature loading most on PC2 (Fig. 6). In the PCA biplot, grids from the Middle zone span the widest range and display the largest bubbles, indicating both greater variability in the selected 3D metrics and higher surfer activity. Together, these patterns suggest that sections of reef with rougher and more variably curved structure coincide with greater use, consistent with work showing that reef morphology can shape shoaling, refraction, and breaking processes^16,34^ and with studies highlighting geomorphology as a key determinant of wave quality and surfer preference^10,35^. While live coral contributes to the formation of reef structure, our results point to underlying geomorphology, particularly curvature and surface ruggedness, as more closely aligned spatial concentration of surfing activity at Cloudbreak.

Boosted regression trees are useful here because they model nonlinear responses and simple interactions without assuming a fixed functional form, which suits complex reef topography. In our cross validated model, the relative influence rankings were surface complexity 29.4%, mean slope 24.1%, mean profile curvature 16.1%, mean planform curvature 15.2%, and mean VRM 15.1%. The partial dependence plots for PC1 of surfer activity (Fig. 7) show clear and interpretable thresholds rather than smooth linear trends. PC1 of surfer activity increases sharply once surface complexity passes about the mid-range of observed values and then plateaus, suggesting a minimum structural texture needed before surfer activity increases. Slope shows a similar step increase near moderate inclinations, consistent with steeper faces supporting more surfable sections. Profile curvature contributes little when negative but rises rapidly as values become positive, indicating that positive profile curvature, meaning convex features along the slope, is associated with higher activity. Planform curvature is broadly positive with a narrow dip around values near zero, consistent with side-to-side undulations that define spur and groove fields being more favorable than areas with little side to side undulation. VRM shows a clear threshold where activity increases quickly once ruggedness exceeds a moderate level. Together, these patterns show that surfer activity concentrates where structural conditions fall within specific ranges of complexity, slope, curvature, and ruggedness, rather than simply increasing with overall structure. We interpret these as associations rather than causal effects, but they underscore the value of high-resolution topography for surf break studies and suggest that metrics such as VRM and curvature can inform surf resource management, artificial reef design, and conservation planning in wave dependent coastal settings.

The findings of this study open several avenues for future research. While GPS-based surfer data provided valuable insight into spatial use patterns, integrating additional environmental parameters, such as wave height, swell direction, and current velocity would allow for a more comprehensive understanding of how physical oceanographic processes interact with reef structure to shape surfing experiences. Although this study revealed strong associations between surfer behavior and structural complexity metrics like VRM and curvature, the absence of direct wave energy measurements limits the ability to fully disentangle the relative contributions of hydrodynamic versus topographic factors. Longitudinal data collection across multiple swell events or seasons could also help capture temporal variability in surfer behavior, reef usage, and potential shifts in human–environment interactions under different oceanographic conditions.

This study also demonstrates the untapped potential of surfers as passive data collectors in high-energy reef environments. GPS-based tracking has been widely used to study human activity patterns in urban and terrestrial environments^15,36^, but its application to marine recreational settings remains limited. Unlike conventional scientific surveys, which are often restricted to low-energy conditions for safety and logistical reasons, surfers are actively present during extreme wave events precisely when the most dynamic reef–wave interactions occur. The GPS tracking data analyzed here were collected during natural recreational behavior and provided detailed spatial patterns of reef use that would have been difficult to obtain through direct scientific observation, especially under hazardous swell conditions. This approach offers a powerful model for participatory marine science, particularly in environments where traditional monitoring efforts are constrained. Expanding this approach to other surf breaks with varying geomorphology, wave exposure, and levels of human use would allow for assessment of the generality of these findings and further refine our understanding of how human activities interact with the physical and ecological dynamics of coral reef ecosystems.

From a management perspective, these results highlight the importance of preserving the physical complexity of coral reefs, particularly in high-use recreational areas. Managers could prioritize actions that avoid smoothing of reef relief, minimize activities that reduce fine scale structure, and place moorings and access routes to steer use away from the most sensitive structural features. Structural features such as concave reef forms and high surface roughness contribute not only to ecological resilience^37^ but also to the maintenance of surfable wave conditions valued by coastal communities and tourism economies^6,16^. As climate change, coastal development, and reef degradation continue to threaten reef systems globally^13,38^, maintaining or restoring fine-scale topographic complexity may be critical for sustaining both ecological function and the cultural and economic benefits associated with surf breaks^1,2^. Moreover, crowd sourced GPS tracking provides a scalable and cost-effective approach for monitoring human interactions with marine ecosystems^15,36^, particularly in high energy or remote locations where conventional scientific surveys are constrained^17^. Integrating these participatory monitoring techniques into conservation initiatives and surf tourism management plans can improve zoning decisions, track changes through time, and align visitor use with habitat protection to safeguard both reef habitats and the socio-economic services they provide.

## Supplementary Information

Below is the link to the electronic supplementary material.

Supplementary Material 1

## Data Availability

The datasets used and/or analyzed during the current study available from the correspoonding author on reasonable request.
