# Supplementary material for: Mapping the relationship of reef structure and surfer spatial patterns at Cloudbreak, Fiji
Source: Sci Rep. 2025 Dec 4;16:1147. doi: 10.1038/s41598-025-30878-6 (PMC12789090; doi:10.1038/s41598-025-30878-6)
Supplement: Supplementary file 1 — Supplementary Material 1 [file 41598_2025_30878_MOESM1_ESM.pdf]

Supplementary Information

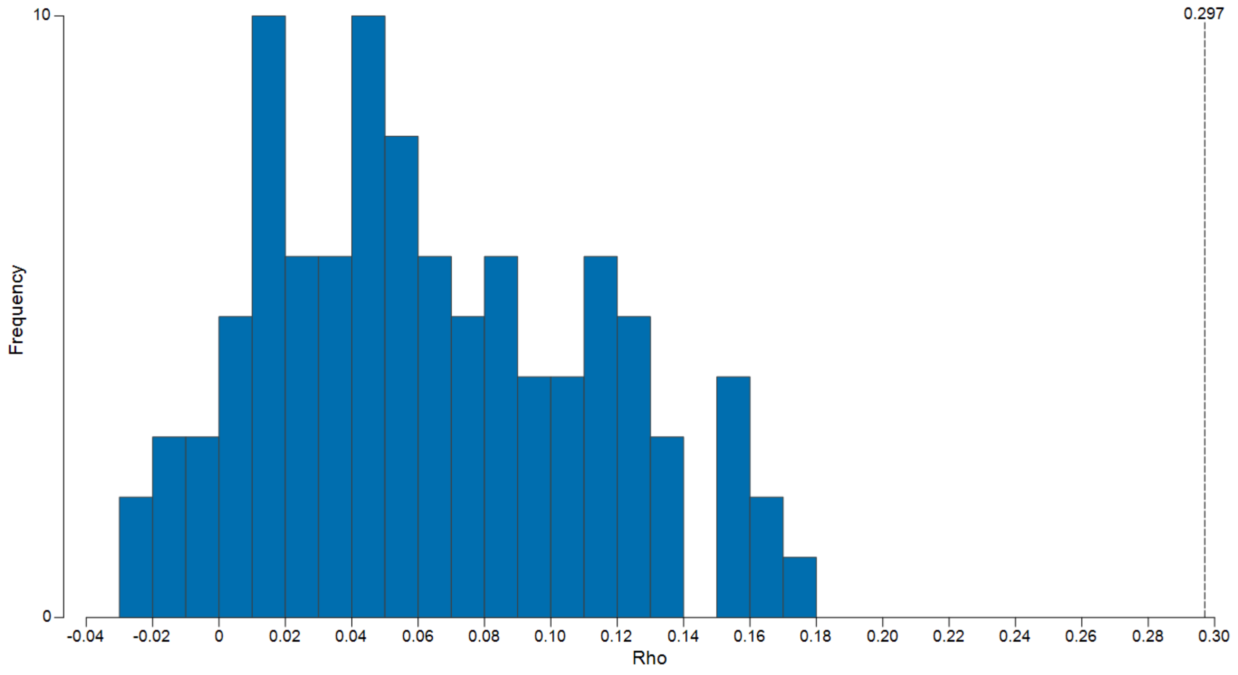

Figure S1. Histogram showing the distribution of correlation values (Rho) generated from 99 randomized permutations of the surfer resemblance matrix used to assess the relationship with 3D habitat metrics (mean VRM, mean curvature, mean planform curvature, and surface complexity). The vertical dashed line represents the observed correlation coefficient (Rho = 0.297), which exceeded all randomized values (p = 0.01), indicating a statistically significant association between surfer spatial patterns and habitat complexity.

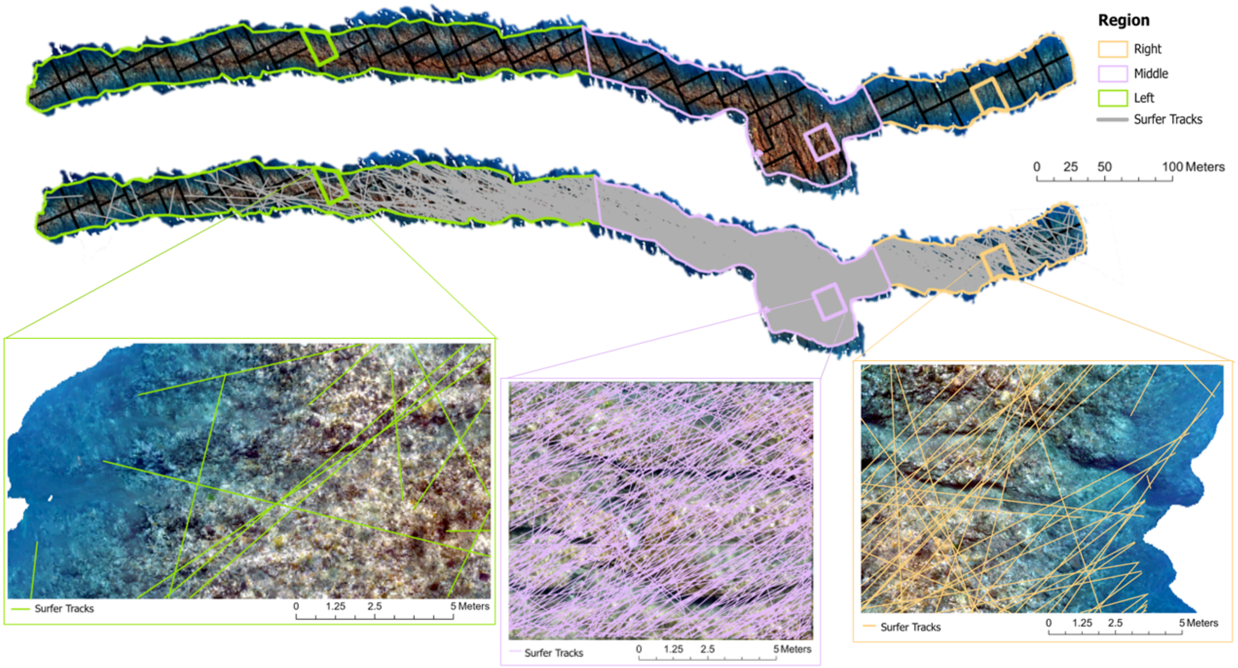

Figure S2. Orthomosaic map and surfer track overlay of the Cloudbreak reef, Fiji. The top panel displays the segmentation of the reef into three distinct regions: Left (green outline), Middle (purple outline), and Right (yellow outline), with black grid overlays delineating subsampled plots used for analysis. Surfer tracks are indicated by thin gray lines, demonstrating spatial patterns of surfer movement across the reef zones. The bottom panels provide detailed insets of surfer track density within each region, highlighting differences in track coverage and surfer behavior.
